# Bridging the gap – MTI experience to an excellent MTI experience – An experiential, theme driven, concordance verifying study

**DOI:** 10.1192/bjo.2021.385

**Published:** 2021-06-18

**Authors:** Arun Enara, Kabir Garg, Ramachandran Kanchana

**Affiliations:** 1 Hertfordshire partnership Foundation NHS trust; 2 Oxleas NHS Foundation Trust

## Abstract

**Aims:**

To collate experiences of international medical graduates (trained psychiatrists) on the Medical Training Initiative (MTI) and equivalent programs (International Medical Fellowship (IMF)/CESR Fellowships) in the United Kingdom and to understand shared themes.

**Method:**

Three psychiatrists with the experience of being part of MTI/IMF program, for a minimum of 1 year, participated in theme guided, focussed discussions to understand common experiences. These discussion where limited to 3 broad headings. Opportunities to grow, what we wish the college knew and what we wished the trusts and supervisors knew. The experiential accounts were captured and circulated among a group of 20 MTI/IMF/CESR fellowship doctors and rated on a 5 point Likert scale varying between strongly agree to strongly disagree.

**Result:**

The findings suggest that the expectations and experiences of the psychiatrists on such programs share some common themes. Most of them had varied experiences under the theme ‘opportunities to grow’. The suggestions for what these doctors ‘wished the trusts, college and supervisors knew’ had a good concordance among the 20 doctors who reviewed the themes and suggestions. The details of the themes and commonalities will be discussed at the conference.

**Conclusion:**

The expectations and experiences of the doctors on MTI/equivalent program share common themes. Bridging the gap between MTI experience to an excellent MTI experience would involve identifying such shared experiences, that could potentially guide development of processes, thereby making these training fellowships better tailored to each trainee.

